# A Fatal Case of Drug-Induced Lung Injury Potentially Related to Olanzapine

**DOI:** 10.7759/cureus.57571

**Published:** 2024-04-03

**Authors:** Tomohiro Akaba, Yuno Shiota, Azusa MIyoshi, Etsuko Tagaya

**Affiliations:** 1 Department of Respiratory Medicine, Tokyo Women's Medical University, Tokyo, JPN

**Keywords:** pulmonary alveolar hemorrhage, steroid, psychiatric disorder, olanzapine, drug-induced lung injury

## Abstract

Drug-induced lung injury (DILI) occurs when exposure to a drug leads to inflammation and, eventually, fibrosis of the lung interstitium. While DILI is a rare side effect of antipsychotic medication, once it manifests, it requires detailed investigation and prompt treatment. Diagnosing DILI can be challenging at times due to its similarity to conditions such as infectious diseases or interstitial pneumonia induced by other causes. We hereby report a fatal case of suspected DILI associated with olanzapine. A 61-year-old female with a history of delusional disorder was admitted to our hospital due to worsened psychiatric symptoms. Ten milligrams of olanzapine had been initiated a week prior to admission by a psychiatrist at the local clinic to control these symptoms. After admission, although the patient claimed no respiratory symptoms, she developed a slight fever and deterioration of chest radiologic findings. Bronchoalveolar lavage revealed a progressively bloody return of fluid, suggesting pulmonary alveolar hemorrhage. Since no respiratory disorders have been noted, and considering the exclusion of other potential diagnoses, DILI was strongly suspected. Although olanzapine was promptly discontinued, the patient's condition rapidly deteriorated. Despite high-dose steroid therapy, the patient's response to treatment was inadequate, and she finally succumbed to the illness. This case highlights that olanzapine may induce lung injury similar to other psychiatric drugs. Furthermore, early diagnosis and treatment are essential for patients with psychiatric disorders who may sometimes present with fewer symptoms.

## Introduction

Common side effects associated with medications, in general, include skin rash, liver damage, and kidney dysfunction. Although drug-induced lung injury (DILI) is a relatively rare side effect, once it occurs, it necessitates the discontinuation of medication and the initiation of immunosuppressive treatment [1]. Moreover, severe cases of DILI can lead to respiratory failure and, sometimes, life-threatening situations, even with intensive treatment. Although the detailed mechanism of drug-induced lung injury development is not well understood, it can occur through two possible mechanisms: direct cytotoxic effects (resulting from drug accumulation due to the drug itself, interactions with other drugs, or abnormal metabolism) and indirect cytotoxic effects (such as allergic reactions).

Olanzapine is a commonly used medication in the field of psychiatry for conditions such as schizophrenia and bipolar disorder. Extensive research has been conducted on its efficacy and safety [2]. It is also used outside of psychiatric diseases, such as in the prevention of chemotherapy-induced nausea and vomiting [3]. Olanzapine exerts its effects by blocking multiple neurotransmitters. Specifically, it acts on dopamine receptors at D1, D2, D3, and D4; serotonin receptors at 5-HT type 2a, 5-HT type 2c, 5-HT3, and 5-HT type 6; alpha1-adrenergic receptors for catecholamines; muscarinic receptors for acetylcholine; and H1 receptors for histamine in the central nervous system [4]. The main reported side effects include weight gain, drowsiness, insomnia, constipation, akathisia, and increased appetite [5]. However, to date, olanzapine-related pulmonary complications have been rarely reported [6].

Here, we present a fatal case of DILI potentially related to olanzapine. While there have been no previous reports of olanzapine-induced lung injury leading to fatal outcomes, our present case marks the first documented instance of such an association.

## Case presentation

A 61-year-old woman with a history of delusional disorder spanning over 20 years was admitted to the psychiatry department due to the worsening of persecution symptoms. She had started taking 10 mg of olanzapine to manage her symptoms one week prior to admission under the care of a psychiatrist at the local clinic to control these symptoms. The patient's medical history included hypertension and chronic kidney disease, but no prior lung conditions. She smoked 20 cigarettes per day until age 55 and had allergies to carbamazepine and valproic acid. She was unemployed, with no history of occupational dust exposure or pet ownership. Additionally, the patient did not use any supplements or illegal drugs.

**Figure 1:**
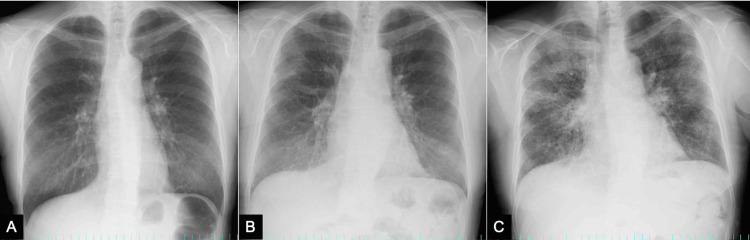
A series of chest X-rays (A) Chest X-ray one year before admission, (B) Chest X-ray on admission showing bilateral peripheral ground-glass opacities, (C) Chest X-ray on day nine showing the deterioration of lung opacities

Upon admission, the patient presented with a low-grade fever and swelling of the left ankle, with no respiratory symptoms reported. The patient's oxygen saturation remained at 95% on room air. A chest X-ray taken one year prior to admission showed no significant abnormalities; however, bilateral peripheral ground-glass opacities were observed on admission (Figure 1).

Because the patient had injured her leg four days before admission, she was started on cefazoline for cellulitis treatment. Swelling of the left ankle improved after the treatment. However, due to persistent fever and elevated C-reactive protein levels (CRP) with limited improvement, piperacillin-tazobactam was initiated instead of cefazoline on day seven. Although the patient denied respiratory symptoms, an increasing oxygen demand was noted (oxygen saturation 92% on 2 L/min). Subsequently, she was transferred to the pulmonology department for further evaluation on day nine.

When transferred to the pulmonary department, fine crackles were detected in lung sounds. While no specific abnormalities were noted in heart sounds, there were also no abnormalities detected in the abdomen, extremities, or neurology. Blood tests showed negative results for all markers related to connective tissue diseases or infectious diseases (Table 1).

**Table 1:** Laboratory findings when transferred to the pulmonary department WBC - white blood cell; RBC - red blood cell; AST - aspartate transferase; ALT - alanine transaminase; LDH - lactate dehydrogenase; γGTP - gamma-glutamyl transpeptidase; BUN - blood urea nitrogen; CRP - C-reactive protein; KL-6 - krebs von den lungen 6; SSA - Sjögren's-syndrome-related antigen A; SSB - Sjögren's-syndrome-related antigen B; MPO-ANCA - myeloperoxidase-anti-neutrophil cytoplasmic antibody; PR3-ANCA - proteinase 3-anti-neutrophil cytoplasmic antibody; GBM - glomerular basement membrane; CMV - cytomegarovirus; TB - tuberculosis

Investigation	Result	Reference value
WBC (/μL)	12080	4000 ~ 8600
Neutrophil (%)	77.3	38 ~ 70
Lymphocyte (%)	10.7	27 ~ 45
Monocyte (%)	7.3	0 ~ 7
Eosinophil (%)	4.5	0 ~ 2
RBC (/μL)	3.43 × 10*6	3.80 × 10*6 ~ 4.80 × 10*6
Hemoglobin (g/dL)	10.1	12.0 ~ 16.0
Hematocrit (%)	31.9	35.0 ~ 43.0
Platelet (/μL)	58.5 × 10*4	15.0 × 10*4 ~ 35.0 × 10*4
Total protein (g/dL)	7.5	6.5 ~ 8.2
Albumin (g/dL)	3.1	3.8 ~ 5.1
Total bilirubin (mg/dL)	0.6	0.2 ~ 1.2
AST (U/L)	33	13 ~ 33
ALT (U/L)	11	6 ~ 31
LDH (U/L)	431	119 ~ 229
γ-GTP (U/L)	64	6 ~ 46
Creatinine (mg/dL)	0.75	0.48 ~ 0.79
BUN (mg/dL)	9.5	8.0 ~ 20.0
CRP (mg/dL)	10.88	0 ~ 0.30
KL-6 (U/mL)	451	< 500
SP-D (ng/mL)	126	< 110
Antinuclear antibody	< 40	< 40
Rheumatoid factor (U/mL)	< 5	< 15
Anti-Ro (SSA) antibody (U/mL)	< 7.0	< 7.0
Anti-La (SSB) antibody (U/mL)	< 7.0	< 7.0
MPO-ANCA (U/mL)	< 1.0	< 1.0
PR3-ANCA (U/mL)	< 1.0	< 1.0
Anti GBM antibody (U/mL)	< 2.0	< 2.0
β-D glucan (pg/mL)	19.2	< 20
CMV antigenemia	negative	negative
T-SPOT.TB	negative	negative
Pneumococcal urine antigen test	negative	negative
Legionella urine antigen test	negative	negative

**Figure 2:**
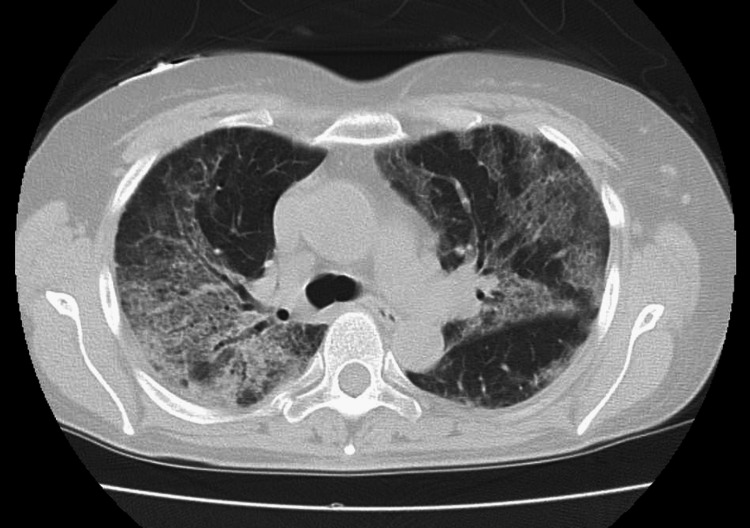
Chest computed tomography on day nine Chest computed tomography showing the diffuse ground-glass opacities and tracheal bronchial ectasis in both lungs

Both blood and sputum cultures returned negative results. A chest computed tomography scan revealed ground-glass opacities in both lungs, indicating a diffuse alveolar damage pattern (Figure 2).

Despite antibiotic therapy, the patient exhibited a poor response. A chest X-ray showed further deterioration of the lungs, and oxygen demands increased to 4 L/min. Consequently, bronchoalveolar lavage was performed for investigative purposes on day 10. The examination revealed an increase in neutrophils, eosinophils, and lymphocytes (Table 2).

**Table 2:** The result of bronchoalveolar lavage fluid CD - cluster of differentiation

Investigation	Result	Reference value
Total cell counts (/mL)	1.75×10*5	0.5×10*5 ~ 2.0×10*5
Macrophage (%)	43.9	80 ~ 94
Lymphocyte (%)	28.4	5.0 ~ 18
Neutrophil (%)	18.1	0 ~ 2.0
Eosinophil (%)	8	0 ~ 1.0
Basophil (%)	0.8	0
Mast cell (%)	0.8	0
CD4/CD8 ratio	2.95	1.0 ~ 2.0

**Figure 3:**
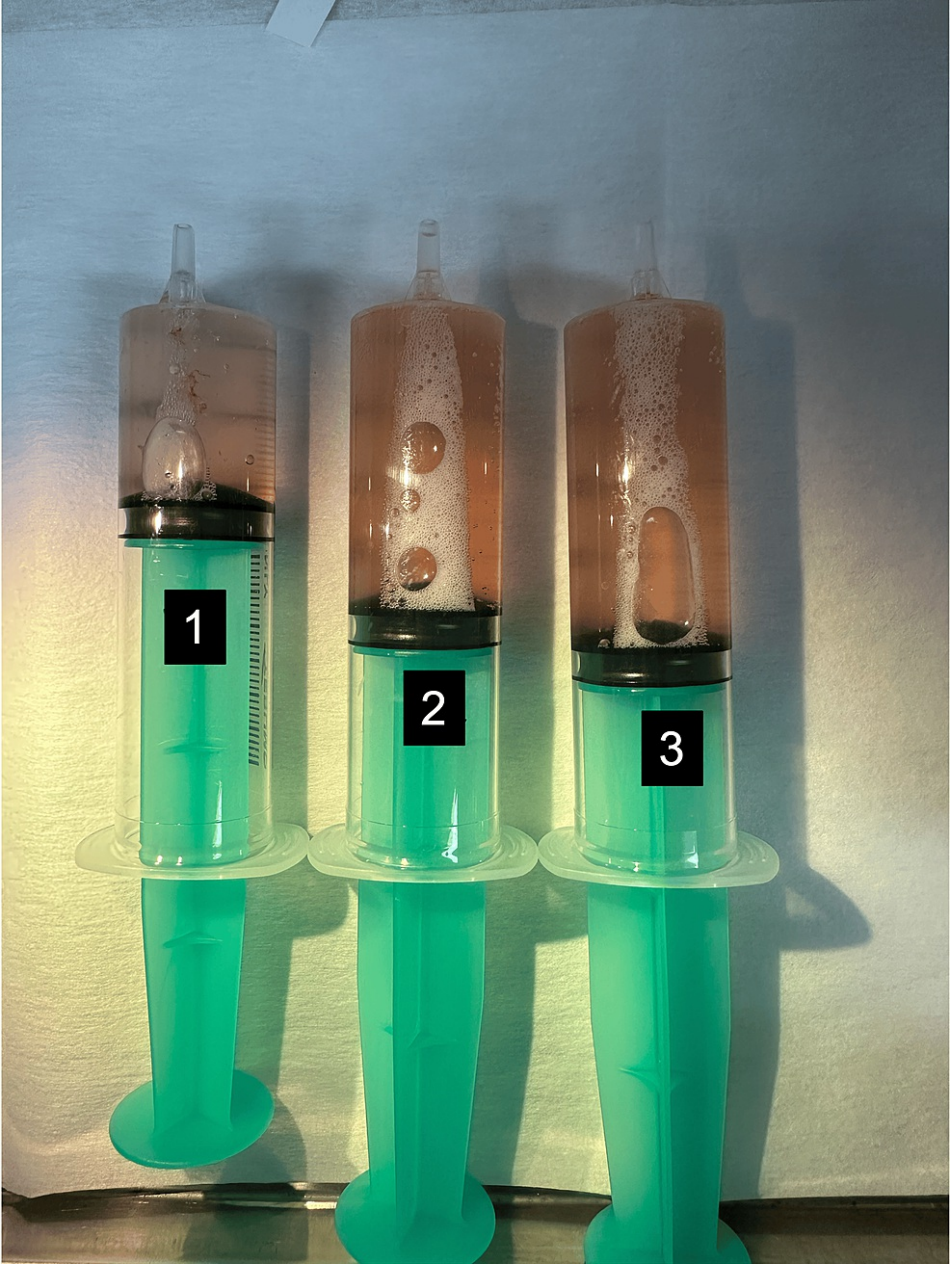
The appearance of bronchoalveolar lavage fluid A progressively bloody return of fluid (from left to right)

The culture for bronchoalveolar fluid was negative. Moreover, a progressively bloody return of fluid was seen, suggesting pulmonary alveolar hemorrhage (Figure 3).

Although the drug-induced lymphocyte stimulation test (DLST) for olanzapine yielded negative results, due to suspicion of drug-induced lung injury (DILI) arising from the clinical course and laboratory findings, olanzapine was discontinued. Additionally, high-dose steroid therapy (1000 mg/day of methylprednisolone for three days and subsequent treatment of 60 mg/day of prednisolone) was initiated; however, the patient showed a limited response to treatment and progressively deteriorated, ultimately resulting in death confirmed on day 18.

## Discussion

DILI can occur with any medication and at any time; therefore, the diagnosis is mainly made by the clinical history and meticulous exclusion of all other possible causes [7]. Because all types of drugs may cause DILI, we should always consider this as a potential differential diagnosis. DLST can be a useful tool for diagnosing DILI. However, DLST results are not always positive, especially during the acute phase of DILI [8]. Although the DLST for olanzapine was negative, we, therefore, made the diagnosis of DILI based on the exclusion of other possible causes, such as bacterial infection, interstitial pneumonia associated with connective tissue diseases, or acute respiratory distress syndrome secondary to cellulitis. Additionally, considering that smoking increases the risk of DILI, the patient's long history of smoking supported the diagnosis of DILI [1]. The absence of prior reports leading to a fatal outcome highlights the importance of recognizing the potential adverse effects of olanzapine. While the literature has documented pulmonary complications associated with the same class of medication, quetiapine, our case suggests a need for increased vigilance regarding olanzapine use as well [9].

There are several types of chest imaging findings for DILI: organizing pneumonia pattern, non-specific interstitial pneumonia pattern, hypersensitive pneumonitis pattern, and diffuse alveolar damage pattern [1, 10]. Among them, the diffuse alveolar damage pattern showed a poor prognosis even in intensive care, including high-dose steroids [11]. Hence, prompt diagnosis and initiation of treatment are crucial upon suspicion of this pattern. In this case, although we discontinued olanzapine and started high-dose steroids immediately after suspecting DILI, the patient unfortunately did not respond to the treatment. Immunosuppressive agents, intravenous immunoglobulin, and/or plasma exchange therapy showed effectiveness for steroid-resistant DILI in previous reports [12]. However, further study is required to reach definitive conclusions.

From the present case, it is important to emphasize that patients with psychiatric disorders may not always exhibit typical respiratory symptoms, even as lung injury progresses, leading to delays in treatment intervention. This phenomenon could be attributed to the fact that patients with psychiatric disorders may report fewer symptoms owing to the nature of the illness itself and the sedative drugs used in treatment. Previous studies have indicated that individuals with mental disorders often have poorer prognoses, partly due to delays in intervention [13]. Similarly, our patient did not report any respiratory symptoms upon admission, which led to a delayed diagnosis of DILI and subsequent poor clinical outcomes. As a point of reflection, considering that the chest X-ray upon admission revealed lung deterioration in this case, the earlier investigation should have been considered even though the patient did not report any respiratory symptoms. Therefore, clinicians should consider DILI in the differential diagnosis of psychiatric patients receiving antipsychotic medications, particularly those showing signs of respiratory deterioration.

## Conclusions

This case report underscores the potential for olanzapine-induced lung injury, a rare yet critical adverse effect warranting attention. All medications carry the potential to cause DILI. When DILI is suspected, prompt discontinuation of the suspected drug and intervention are crucial, especially if radiographic findings indicate a diffuse alveolar damage pattern. While evidence regarding the treatment of severe DILI is limited, intensive therapies such as high-dose steroid therapy, immunosuppressive agents, intravenous immunoglobulin, and/or plasma exchange therapy may be viable options.

Furthermore, because patients with psychiatric disorders may exhibit fewer symptoms despite disease progression, our case underscores the importance of recognizing subtle symptom presentations in such patients. Failure to promptly detect these symptoms can lead to delayed intervention and fatal outcomes.
